# The association between self-reported mental health, medication record and suicide risk: A population wide study

**DOI:** 10.1016/j.ssmph.2021.100749

**Published:** 2021-02-02

**Authors:** Ifeoma N. Onyeka, Dermot O’Reilly, Aideen Maguire

**Affiliations:** a Centre for Public Health, Queen's University Belfast, Royal Hospitals Site, Grosvenor Road, Belfast, UK; b Administrative Data Research Centre Northern Ireland, Centre for Public Health, Queen's University Belfast, Royal Hospitals Site, Grosvenor Road, Belfast, UK

**Keywords:** Suicide, Mental health, Psychotropic medication

## Abstract

Suicide mortality and mental ill health are increasing globally. Mental ill health can be measured in multiple ways. It is unclear which measure is most associated with suicide risk. This study explored the association between self-rated mental health and medication record and death by suicide. The 2011 Northern Ireland Census records of adults aged 18-74 years (n=1,098,967) were linked to a centralised database of dispensed prescription medication and death registrations until the end of 2015. Mental health status was ascertained through both a single-item self-reported question in the Census and receipt of psychotropic medication. Logistic regression models examined the association between indicators of mental ill health and likelihood of suicide mortality. Of the 1,098,967 cohort members, 857 died by suicide during the study period. Just over half of these deaths (n=429, 50.1%) occurred in individuals with neither indicator of mental ill health. Cohort members with both self-reported mental ill health and receipt of psychotropic medication had the highest risk of suicide (OR=6.13, 95%CI: 4.94–7.61), followed by those with psychotropic medication record only (OR=4.00, 95%CI: 3.28–4.88) and self-report only (OR=2.88, 95%CI: 2.16–3.84). Individuals who report mental ill health and have a history of psychotropic medication use are at a high risk of suicide mortality. However, neither measure is particularly sensitive, as both failed to signal over half of subsequent suicides. Some individuals who report poor mental health but are not in receipt of psychotropic medication are at increased risk of suicide, indicating possible unmet treatment need. The combination of the two indicators offers more precision for identifying those most at risk for targeted interventions.

## Highlights

• Mental ill health is associated with an increased risk of suicide, but measuring population mental health is difficult.
• The suicide risk associated with both subjective and objective indicators of mental ill health have not been examined.
• Most individuals who die by suicide have no indicator of mental ill health.
• Having both self-reported poor mental health and psychotropic medication record carried the highest risk of suicide death.
• Increased risk of suicide in individuals who report poor mental health but are not on medication may indicate unmet need.

## 1 Introduction

Death by suicide is a major public health concern. It is estimated that worldwide there are over 800,000 suicides each year with an age-standardised suicide rate of approximately 11.4 per 100,000 population (World Health Organization [WHO] 2014). In the UK, the suicide rate in 2015 was 10.9 per 100,000, with the highest rate observed in Northern Ireland at 17.2 per 100,000 (Scowcroft, 2017). Previous research suggests that death by suicide is most common in men, in mid-life/old age, and in individuals with low socioeconomic status and with comorbid physical health conditions (Almeida et al., 2016; Batty et al., 2018; Burns, 2016; Knipe et al., 2015; Korkeila et al., 2007). Wider societal impacts are even greater due to the effects on friends, family members, and colleagues of the deceased (Gunnell, 2015). Effective identification and treatment amongst those most at risk is necessary to reduce the burden of suicide. It is therefore of paramount importance to better identify those most at risk.

Rates of mental ill health are also increasing, with mental health conditions predicted to become the leading cause of burden-of-disease in developed countries by 2030 (Mathers & Loncar, 2006). The number of individuals in receipt of psychotropic medications has also increased in parallel (Chien et al., 2007; Ilyas & Moncrieff, 2012; Pratt et al., 2011; Steinhausen, 2015; Stephenson et al., 2013). Although poor mental health is a recognised major risk factor for death by suicide (Tong & Phillips, 2010), identifying people in the population with mental ill health can be difficult. Less than a third of individuals who die by suicide have had contact with mental health services in the 12 months before their death (NCISH 2018; Stene-Larsen & Reneflot, 2019), and a recent review of suicide research in Northern Ireland has highlighted the need for population level interventions and interventions for individuals in high risk groups (O’Neill & O’Connor 2020). Therefore, there is a clear need to identify those who may be in high risk groups using routinely collected mental health indicators. Self-reported mental ill health, and records of dispensed psychotropic medication are two commonly used proxy indicators of mental ill health. Understanding which indicators of mental ill health are most associated with the risk of death by suicide could be used to inform future studies on suicide risk and help identify groups for targeted interventions.

Self-reported mental ill health based on a single item question is popular in research and in primary care settings (McAlpine et al., 2018; Mitchell et al., 2011). A review of 57 studies (Ahmad et al., 2014) has highlighted the utility of one single self-reported mental health question as a robust mental health indicator with the potential to be used to predict future mental health outcomes, health service utilisation and service satisfaction. A large-scale study of 131,362 individuals in Norway, found a dose-response association between severity of self-reported mental health and risk of death by suicide (Bramness et al., 2010) and Gunnell and colleagues in the UK, found a six-fold increase in the 20-year suicide risk in men who reported minor mental disorder compared to those who did not (Gunnell et al., 2002). Both studies were limited by the inclusion of only middle-aged/older adults, and relatively small numbers of outcomes. However, measures of self-reported mental health are not generally available at a population-wide level and so data relating to prescribed medication used for treatment of mental health conditions are increasingly being used as proxy indicators. Such data are available at a population level especially in the UK and Nordic countries, even though register-type data can be sometimes difficult and costly to access (Bosqui et al., 2019; Doebler et al., 2017; Henriksson et al., 2003; Maguire et al., 2016; Straiton et al., 2017).

That individuals in receipt of psychotropic medications are at increased risk of suicide has been demonstrated in Norway, Japan and England (Reneflot et al., 2019; Takeuchi et al., 2017; Windfuhr et al., 2016). Studies have shown that 36–51.7% of suicide decedents were in receipt of prescriptions for psychotropic medication 3 to 12 months before their death (Appleby et al., 2014; Benson et al., 2018; Henriksson et al., 2001) and a post-mortem toxicological analysis found evidence of psychotropic medication use in nearly half (45.3%) of 5,281 suicides in Sweden (Isacsson et al., 1999). It has therefore been suggested that information on psychotropic medication use has a potential role in suicide risk prediction (Choi et al., 2018).

However, a direct comparison between self-reported mental ill health and records of dispensed psychotropic medication, and suicide has not been reported to date. The few existing general population studies tend to investigate these indicators separately, rather than in combination. Therefore the relative utility of these two indicators of mental ill health as a measure of suicide risk remains unclear. One of these two indicators, or both combined, might offer better precision for identifying individuals at risk of dying by suicide. Although it has been recommend that risk assessments are not to be used alone to inform treatment strategies or predict future risk of suicide (NICE 2011; Steeg et al., 2018), understanding populations most at risk is important for targeting and evaluating interventions. This population-wide study will use administrative data on over one million Northern Ireland adult residents to assess the relationship between self-reported mental ill health, psychotropic medication record and both in combination, and subsequent death by suicide.

## 2 Methods

This data linkage study involved linking anonymised data from several sources on all individuals aged 18-74 years living in Northern Ireland. First, data from the most recent Census conducted in March 2011; second, psychotropic medication data from the Enhanced Prescribing Database (EPD), a nationwide centralised electronic database of all medications dispensed in community pharmacies throughout the country; and lastly, death registrations from the General Register Office.

### 2.1 Self-reported mental ill health

Self-reported mental ill health was derived from responses to a Census question that asked respondents if they had experienced “*an emotional, psychological or mental health condition (such as depression or schizophrenia) which has lasted, or was expected to last at least* 12 months”. Affirmative responses were deemed a proxy for poor self-reported mental health. Previous studies have used this as an indicator of mental ill health in young people, caregivers, minority groups, and individuals exposed to environmental influences (Tseliou et al., 2016; Maguire et al., 2017; Doebler et al., 2017; Bosqui et al., 2017; Wright et al., 2018; Rosato et al., 2019).

### 2.2 Medication record

Northern Ireland has free-at-the-point-of-service national health care that includes free prescriptions. The EPD contains information on all prescriptions dispensed by community pharmacies. For this study, the information of interest was a record of psychotropic medication. This was categorised using the British National Formulary (BNF), a standard nomenclature for medications in the UK (BNF 2018), into the following groups: antidepressants (BNF 4.3.1, 4.3.2, 4.3.3, 4.3.4), hypnotics (BNF 4.1.1 or 4.1.3), anxiolytics (BNF 4.1.2), antipsychotics (BNF 4.2.1 or 4.2.2) and antimania medication (BNF 4.2.3). Individuals were considered to be in receipt of psychotropic medication if they had two or more records of any psychotropic medication in the five months around Census (i.e. January, February, March, April, May). “No use” means zero medication or a one-off/singular prescription. This was premised on the fact that individuals with two or more repeat prescriptions were likely to be those experiencing long-term mental health problems and excludes once/one-off prescriptions that might be for a transient event. This approach has been used in previous studies (Kessing et al., 2005; Maguire et al., 2016).

### 2.3 Suicide data

Mortality data from the General Register Office from Census month (March 2011) to December 2015 were then linked to Census records to estimate mortality risk in the following 57 months. This linkage process has been described previously (O’Reilly et al., 2008). The primary outcome of interest was death by suicide, which was defined as main causes of death with International Classification of Disease version 10 (ICD-10) codes X60–X84, Y10-Y34, and Y870 denoting both definite suicide and those of undetermined intent as per usual practice.

### 2.4 Covariates

The Census data contains a wealth of sociodemographic and household information and area-level attributes in addition to self-reported mental and physical health. Covariates were selected based on known association to either mental health or death by suicide.

#### 2.4.1 Sociodemographic characteristics

These included age, gender, marital status (categorised as never married; married or co-habiting; separated, widowed or divorced), religious affiliation (Protestant; Catholic; none/other), whether the person was living in a single-person household and educational attainment (no formal qualifications; basic; A-levels; first degree or higher). Low social economic status and religious affiliation are associated with suicide (Batty et al., 2018; Knipe et al., 2015; O’Reilly & Rosato 2015; Spoerri et al., 2010).

#### 2.4.2 Housing tenure/value

Measures of the capital value of a house and housing tenure were combined to derive an eight-fold classification of tenure/rateable value of property: private renting; social renting; and, for owner-occupiers, six categories of house value ranging from less than £75,000 to over £200,000, with a separate category for owners with homes as yet unvalued. This housing tenure/house value variable has been used before as an effective indicator of cumulative wealth and it correlates with health status and predicts future mortality (Bosqui et al., 2017; Connolly et al., 2010; O’Reilly et al., 2015).

#### 2.4.3 Area-level attributes

Two characteristics of area of residence were also included; the first an indicator of area deprivation was derived from the income domain of the Northern Ireland Index of Multiple Deprivation ranked from least to most deprived and grouped into quintiles (Northern Ireland Statistics and Research Agency [NISRA] 2010). The second was an indicator of urban/rural residence based on an official classification of settlements (NISRA 2015) and grouped as urban, intermediate and rural. Area of residence and neighbourhood deprivation have been shown to be associated with death by suicide (Batty et al., 2018; Qin, 2005).

#### 2.4.4 Physical health condition

Physical health was defined in two ways: the number of physical health conditions and the presence of activity limitation. An item in the Census questionnaire asked participants whether they “have any of the following conditions which have lasted, or are expected to last at least 12 months”, and people could tick all the conditions from a list of conditions that related to them. The presence of two or more chronic conditions was defined as multimorbidity. Another Census item asked “Are your day-to-day activities limited because of a health problem or disability which has lasted, or is expected to last at least 12 months?” with responses, no; yes-limited a little; yes-limited a lot. Suicide is common in those with comorbid multiple physical health conditions and limitations of daily activities (Almeida et al., 2016; Onyeka et al., 2020).

### 2.5 Statistical analysis

STATA 15 software was used for all data analyses. Descriptive statistics were used to analyse demographic and other personal characteristics. A series of individual logistic regression models were generated to examine the relationship of the indicators of mental health status on likelihood of death by suicide. This included (1) those with neither poor self-reported mental health nor record of psychotropic medication, (2) those with poor self-reported mental health only, (3) those with psychotropic medication only, and (4) those with both poor self-reported mental health and a record of psychotropic medication. The models were progressively adjusted for demographic, socioeconomic and physical health variables. Results are presented as odds ratio (OR) with 95% confidence interval (CI). As follow-up was short and the outcome rare, logistic regression was favoured over Cox Regression (Annesi et al., 1989; Callas et al., 1998; Green & Symons, 1983; Ingram & Kleinman, 1989).

### 2.6 Ethics approval

Approval for this study was obtained from the Office for Research Ethics Committees Northern Ireland (ORECNI), approval reference number 16/SC/0241. Informed consent from the subjects was not necessary because only anonymised data were released to the research team. The linked data were anonymised, held in the Administrative Data Research Centre Northern Ireland (ADRCNI) secure data environment within NISRA and made available to the research team for the purpose of this study.

## 3 Results

The study cohort consisted of 1,098,967 individuals aged 18-74 years who were alive and resident in Northern Ireland at the time of the 2011 Census. Individuals living in communal establishments were not included in the cohort as many of their sociodemographic characteristics would not have been relevant. The demographic and socioeconomic profile of the study cohort are presented in Table 1 along with the numbers and proportions reporting poor mental health in the Census or who had been on psychotropic medication in the months close to the Census date.

**Table 1:** Characteristics of the study cohort along with the numbers and proportions reporting poor mental health in the census or who had been on psychotropic medication in the months close to the census date. Data represents Northern Ireland residents aged 18-74 years (N=1,098,967).

Variable	Total population (N= 1,098,967) n (%)	Mental health measures			
		None^¶^ (N=918,819, 83.6%) n (row %)	self-report only (N=31,435, 2.9%) n (row %)	medication^ŧ^ record only (N=94,677, 8.6%) n (row %)	Both self-report & medication (N=54,036, 4.9%) n (row %)
Gender					
Female	570,266 (51.9)	454,875 (79.8)	17,119 (3.0)	64,392 (11.3)	33,880 (5.9)
Male	528,701 (48.1)	463,944 (87.8)	14,316 (2.7)	30,285 (5.7)	20,156 (3.8)
**Age at 2011 Census (years)**					
18–24	138,596 (12.6)	130,747 (94.3)	2,666 (1.9)	3,089 (2.2)	2,094 (1.5)
25–34	205,415 (18.7)	184,293 (89.7)	5,384 (2.6)	9,199 (4.5)	6,539 (3.2)
35–44	222,155 (20.2)	186,045 (83.8)	7,237 (3.3)	16,501 (7.4)	12,372 (5.6)
45–54	224,269 (20.4)	178,332 (79.5)	8,112 (3.6)	21,953 (9.8)	15,872 (7.1)
55–64	176,641 (16.1)	136,756 (77.4)	5,644 (3.2)	22,187 (12.6)	12,054 (6.8)
65–74	131,891 (12.0)	102,646 (77.8)	2,392 (1.8)	21,748 (16.5)	5,105 (3.9)
**Marital status**					
Married/cohabiting	637,732 (58.0)	547,218 (85.8)	12,667 (2.0)	54,568 (8.6)	23,279 (3.7)
Separated/divorced/widowed	141,711 (12.9)	95,584 (67.5)	7,776 (5.5)	22,404 (15.8)	15,947 (11.3)
Never married	319,524 (29.1)	276,017 (86.4)	10,992 (3.4)	17,705 (5.5)	14,810 (4.6)
**Persons in household**					
Others	936,423 (85.2)	802,947 (85.8)	21,866 (2.3)	74,213 (7.9)	37,397 (4.0)
Single-person household	162,544 (14.8)	115,872 (71.3)	9,569 (5.9)	20,464 (12.6)	16,639 (10.2)
**Religion**					
Protestant	473,808 (43.1)	397,922 (84.0)	10,930 (2.3)	42,931 (9.1)	22,025 (4.7)
Catholic	436,358 (39.7)	365,254 (83.7)	12,362 (2.8)	34,583 (7.9)	24,159 (5.5)
Other	188,801 (17.2)	155,643 (82.4)	8,143 (4.3)	17,163 (9.1)	7,852 (4.2)
**Highest qualification**					
1st degree or higher	283,041 (25.8)	254,976 (90.1)	4,609 (1.6)	16,987 (6.0)	6,469 (2.3)
A-levels	144,221 (13.1)	128,950 (89.4)	2,975 (2.1)	8,118 (5.6)	4,178 (2.9)
Basic education	393,097 (35.8)	334,285 (85.0)	11,400 (2.9)	29,939 (7.6)	17,473 (4.4)
No qualification	278,608 (25.4)	200,608 (72.0)	12,451 (4.5)	39,633 (14.2)	25,916 (9.3)
**House tenure/value**					
£200,000+	150,446 (13.7)	136,624 (90.8)	1,991 (1.3)	9,064 (6.0)	2,767 (1.8)
£150,000–199,999	141,679 (12.9)	126,252 (89.1)	2,199 (1.6)	9,869 (7.0)	3,359 (2.4)
£100,000–149,999	250,218 (22.8)	217,377 (86.9)	4,951 (2.0)	19,839 (7.9)	8,051 (3.2)
£75,000–99,999	166,041 (15.1)	139,774 (84.2)	4,155 (2.5)	14,929 (9.0)	7,183 (4.3)
< £75,000	113,057 (10.3)	92,384 (81.7)	3,385 (3.0)	11,668 (10.3)	5,620 (5.0)
Private rent	150,681 (13.7)	121,764 (80.8)	6,501 (4.3)	12,109 (8.0)	10,307 (6.8)
Social rent	126,845 (11.5)	84,644 (66.7)	8,253 (6.5)	17,199 (13.6)	16,749 (13.2)
**Area of residence**					
Rural	297,297 (27.1)	259,783 (87.4)	6,258 (2.1)	21,417 (7.2)	9,839 (3.3)
Intermediate	584,291 (53.2)	485,418 (83.1)	16,507 (2.8)	52,968 (9.1)	29,398 (5.0)
Urban	217,379 (19.8)	173,618 (79.9)	8,670 (4.0)	20,292 (9.3)	14,799 (6.8)
**Income deprivation index quintile**					
1 (least deprived)	213,578 (19.4)	188,405 (88.2)	3,766 (1.8)	15,725 (7.4)	5,682 (2.7)
2	233,004 (21.2)	201,561 (86.5)	5,042 (2.2)	18,084 (7.8)	8,317 (3.6)
3	226,177 (20.5)	192,093 (84.9)	5,596 (2.5)	18,687 (8.3)	9,801 (4.3)
4	220,936 (20.1)	180,189 (81.6)	7,202 (3.3)	20,586 (9.3)	12,959 (5.9)
5 (most deprived)	205,272 (18.7)	156,571 (76.3)	9,829 (4.8)	21,595 (10.5)	17,277 (8.4)
**No. of physical health conditions**					
None or 1	977,341 (88.9)	856,218 (87.6)	19,924 (2.0)	70,708 (7.2)	30,491 (3.1)
2 or more	121,626 (11.1)	62,601 (51.5)	11,511 (9.5)	23,969 (19.7)	23,545 (19.4)
**Limiting long-term illness**					
None	863,897 (78.6)	790,195 (91.5)	9,919 (1.2)	52,515 (6.1)	11,268 (1.3)
A little	102,126 (9.3)	68,065 (66.7)	7,517 (7.4)	14,886 (14.6)	11,658 (11.4)
A lot	132,944 (12.1)	60,559 (45.6)	13,999 (10.5)	27,276 (20.5)	31,110 (23.4)
**Death by suicide**					
No	1,098,110 (99.9)	918,390 (83.6)	31,369 (2.9)	94,522 (8.6)	53,829 (4.9)
Yes	857 (0.08)	429 (50.1)	66 (7.7)	155 (18.1)	207 (24.2)

^**¶**^None; no self-reported poor mental health and ≤1 psychotropic medication record.

^**ŧ**^ Medication **=** psychotropic medication.

The majority of the population had neither indication of mental ill health (83.6%). The overall level of agreement between the two indicators was reflected by a Cohen’s Kappa of 0.40, which is considered borderline fair-moderate. Just over 1 in 13 individuals (7.8%) reported poor mental health in the Census and over 1 in 8 individuals were in receipt of psychotropic medication (13.5%). Overall, 2.9% of the cohort had reported poor mental health but had not received any psychotropic medication, 8.6% had been on psychotropic medication but did not report poor mental health and 4.9% had both poor self-reported mental health and a record of psychotropic medication. A higher proportion of females than males with poor mental health were in receipt of psychotropic medication. The proportion of the population on psychotropic medication rose steeply with age reaching approximately one-in-five over the age of 55 years, which is in stark contrast to the more modest increase in self-reports of poor mental health. About one-third of those who were separated, divorced or widowed had evidence of poor mental health compared to one-in-seven of those who were married. The expected socioeconomic gradients were evident, with, for example, one third of those in social housing reporting either poor mental health or being in receipt of psychotropic medication. The strong relationship between physical and mental health was also clear with approximately half of those with two or more health conditions, or those whose activities were limited a lot, having at least one indicator of poor mental health. A total of 857 individuals died by suicide during the follow-up period, just over half (429) of these occurred in cohort members who had neither reported poor mental health nor had been on psychotropic medication. Of the 148,713 individuals in receipt of medication, 362 died by suicide (0.24%) compared to 495 of the 950,254 individuals not in receipt of medication (0.05%). About one quarter of deaths by suicide occurred in people who had both indicators of poor mental health. One-in-thirteen of suicides occurred in those who had reported poor mental health but who were not on current medication.

Table 2 shows the association between mental health status and the risk of death by suicide (full results available on request). In the unadjusted logistic regression model, those cohort members having both indicators of poor mental health had the highest risk of suicide over the follow-up period (OR=8.23, 95%CI: 6.97–9.72), though this was attenuated to OR=6.13 (95%CI: 4.94–7.61) with further adjustment for other socio-demographic and area-level factors known to increase risk of suicide. In the fully adjusted models, individuals who had been in receipt of psychotropic medication but who had not reported poor mental health in the Census were four times more likely to die by suicide (OR=4.00, 95%CI: 3.28–4.88) compared to those with no indicator of poor mental health. Finally, even those in the smallest group, the 2.9% who reported poor mental health but who had not received any psychotropic medication in the peri-Census period, were almost three times more likely to die by suicide than those with no indicator of poor mental health (OR=2.88, 95%CI: 2.16–3.84).

**Table 2:** The likelihood of death by suicide according to self-reported mental health and record of psychotropic medication, with adjustment for sociodemographic characteristics and physical health conditions. Results show Odds Ratios and (95% Confidence Intervals) from logistic regression models.

Variable	Total population (N= 1,098,967)	Suicide deaths N=857	Unadjusted	Adjusted OR	
				Age & gender	Fully-adjusted *
Mental health status					
No indicator of poor mental health^¶^	918,819 (83.6)	429	1.00 (reference)	1.00 (reference)	1.00 (reference)
Self-reported poor mental health only	31,435 (2.9)	66	4.50 (3.48–5.84)	4.95 (3.81–6.42)	2.88 (2.16–3.84)
Psychotropic medication record only	94,677 (8.6)	155	3.51 (2.92–4.22)	5.38 (4.45–6.51)	4.00 (3.28–4.88)
Both self-report & medication record	54,036 (4.9)	207	8.23 (6.97–9.72)	10.61 (8.94–12.59)	6.13 (4.94–7.61)

OR – odds ratio. CI – confidence interval.

^¶^No indicator; no self-reported poor mental health and ≤1 psychotropic medication record.

*Fully adjusted for age, gender, marital status, single-person household, religion, highest qualification, house tenure/value, area of residence, income deprivation, activity limitation, number of chronic physical health conditions.

## 4 Discussion

This study clearly illustrates that having both a self-report of poor mental health in the Census and being in receipt of psychotropic medication is associated with a high risk of death by suicide, however over half of all suicide deaths had neither indicator of mental ill health. In the study population 7.8% self-reported poor mental health, this is slightly lower than in other population studies (Bramness et al., 2010; Gunnell et al., 2002), but this is likely due to the requirement for chronicity in the Census question. These other population studies used versions of the General Health Questionnaire (GHQ) which asks about mental health status in “the last 2 weeks,” whereas within our study the Census question asked about a mental health condition that had lasted or was expected to last for 12 months. The level of psychotropic medication use (13.5%) was similar to that observed in other Northern Irish studies (Maguire et al., 2016; Tseliou et al., 2018). There was fair-moderate overall agreement in the distribution of the two indicators, which is similar to an earlier report using the GHQ as a measure of psychological well-being compared to psychotropic medication (Tseliou et al., 2018).

To our knowledge this is the first study to directly compare these two indicators of poor mental health. Our findings agree with earlier studies that separately examined self-reported poor mental health (Bramness et al., 2010; Gunnell et al., 2002) and psychotropic medication record (Isacsson et al., 1999; Reneflot et al., 2019; Takeuchi et al., 2017; Windfuhr et al., 2016) and showed them to be important indicators of suicide risk. In our study, the use of two mental health indicators combined (relative to using them separately) offered better precision for identifying individuals at risk of dying by suicide. This may be because this method captures both subjective and objective measures of mental health status. Literature highlighting under-reporting of mental health conditions (Karam et al., 2014) and a need to pair prescription data with other data when being used as proxy for mental health (Gardarsdottir et al., 2007) lend support to this observed precision. Although previous research has looked at receipt of pain medication and mental health medications as predictors of suicide (O’Neill et al., 2019), our paper focuses on mental ill-health as a potential predictor of suicide using two commonly used mental health indicators.

Firstly, the highest likelihood of death by suicide was amongst those who had reported poor mental health in the Census and had been in receipt of psychotropic medication. This may represent both an indication of the severity of the conditions being treated or that the combination of indicators offers more precision; for example, some will have received psychotropic medications for non-mental health issues but this is less likely to be the case for those who also state that they have poor mental health. Having a positive response to both indicators is strongly associated with suicide risk.

Secondly, those in receipt of psychotropic medication only had the next highest likelihood of death by suicide compared to those with no indication of mental ill health. Again, this may represent severity as having a condition that requires medication may be indicative of poorer mental health status compared to someone who does not require medication. Some individuals in receipt of medication did not report poor mental health and this may be due to a range of factors including stigma (Ahmedani, 2011), treatment efficacy, respondents not considering their condition to be chronic, or proxies completing their Census return. Studies considering only one measure of mental ill health should be aware that medication record may be more objective and a better predictor of suicide risk than self-reported mental health. When using receipt of psychotropic medication as proxy indication of suicidality, it is important to note that the direction of the relationship does not imply causation, and caution should be taken not to stigmatise use of psychotropic medication. Primary care is the most predominant health service utilised prior to suicide death (O’Neill et al., 2014). Therefore, individuals who receive psychotropic medications should be assessed for suicidal intent by their General Practitioner, and information should be provided to patients and carers regarding symptoms/suicidal behaviours. This might represent a low-barrier intervention to prevent suicide. Although over 40% of those who die by suicide are in receipt of psychotropic medication, this represents only a small proportion of the population who are in receipt of psychotropic medication and therefore any intervention aimed at this group should not be resource intensive.

Thirdly, over 1 in 13 (7.7%) of the suicides were in individuals who reported poor mental health but did not have any record of receiving psychotropic medication. Self-reported mental ill health is itself a good indicator of suicide risk. Although these individuals may be in receipt of alternative non-pharmacological treatments that were not captured in this study, it is likely that this group may represent either less severe mental health conditions or unmet treatment need. Earlier studies have expressed concerns about treatment rates and unmet need. Findings from the Northern Ireland Study of Health and Stress highlighted substantial delays in seeking mental health treatment: only 48% of those with mood disorders, 16% of those with anxiety and 4% of those with substance use disorders sought treatment within the first year of onset (Bunting et al., 2012).

Lastly, half of those in the cohort who died by suicide had neither reported chronic poor mental health nor were in receipt of psychotropic medication (n=429, 50.1%). Although this is a high proportion, it also means that 49.9% of individuals who died by suicide had an indicator of mental ill health as measured by self-report or medication record. This is higher than the 30% of individuals who die by suicide who had been in contact with mental health care in the 12 months before their death, and highlights not only the difficulty in predicting suicidal behaviour (half of our suicide deaths have no indication of mental ill health) but also in the potential added benefit complementing mental health services data with indicators of self-reported mental health and medication record (NCISH 2018; Stene-Larsen & Reneflot, 2019).

### 4.1 Study strengths and limitations

This is a population-wide study that involved over 1 million residents, and the high number of suicide deaths provided sufficient power for robust estimates. This study extended knowledge by conjointly assessing the relationship of two often used indicators of mental ill health and suicide risk. Unlike existing studies which considered these indicators individually we have directly compared both subjective (self-report) and the arguably more objective records of psychotropic medication use. Self-reports have limitations though it has been argued that these might transcend clinician/interviewer ratings for gathering relevant information in certain situations due to potential bias with the interviewer and a person’s reluctance to disclose a mental illness (Bramness et al., 2010). Using both indicators of mental ill health helped to address shortcomings of the individual indicators – for example, social desirability bias or response bias in self-reports. Although psychotropic medication record provided unbiased information about medication dispensed to the deceased persons, it does not tell us whether the deceased took the medication or not. Although some psychotropic medications are used to treat other non-mental health related disorders, antipsychotic medication is indicative of severe mental illness and the vast majority of antidepressant medication is prescribed for the common mood disorders depression or anxiety (Gardarsdottir et al., 2007). Receipt of psychotropic medication therefore remains an imperfect but invaluable tool in understanding population mental ill health. Further studies are needed to delineate the strengths and weaknesses of these pharmacological proxies for poor mental health.

### 4.2 Policy and practice implications

That half of all the suicide deaths occurred in individuals without either a record of psychotropic medicine or a report of mental ill health highlights the inability of risk indicators to determine suicide at scale. It reinforces the notion that risk assessments are not to be used alone to inform treatment strategies or predict future risk of suicide (NICE 2011; Steeg et al., 2018). Nevertheless, it suggests a need for greater effort to identify and manage mental health problems in order to prevent future deaths. Leavey and colleagues found that 82% of individuals who died by suicide consulted a General Practitioner, 70% for mental health problems, in the 12 months before their death (Leavey et al., 2016). Providing training and support to General Practitioners would be helpful (Hübner-Liebermann et al., 2010; Leavey et al., 2017; Roskar et al., 2010). Future research studies should aim to understand which practical interventions may help prevent suicide deaths among the group who were neither on medications nor reported mental health problems.

Where possible, using a combination of self-report and psychotropic medication record results in the strongest observed associations with death by suicide. This information could be used by individuals attempting to identify those most at risk for targeted interventions. If limited to using one indicator of mental ill health to explore associations with suicide then receipt of psychotropic medication is the indicator of choice. But pairing receipt of psychotropic medication with self-reported poor mental as a proxy for suicidality would be a more practical approach. In this study, persons who had reported poor mental health in the Census and had been in receipt of psychotropic medication had the highest likelihood of suicide death. Being on medication would have been expected to be a protective factor. So elevated suicide risk suggests potentially suboptimal treatment of patients and would require further actions from the healthcare system. For example, regular check-ups, either in person or via telephone, in order to monitor progress and compliance with medication, and to adjust/change medication as needed. As people in this subgroup are likely to have more severe mental health problems, a comprehensive treatment approach incorporating counselling and psychotherapy should be considered. A Swedish study found that individuals on a combination of psychotropic medication with psychotherapy had a lower suicide risk than those on only psychotropic medication (Holländare et al., 2020).

## Author contribution

AM and DOR designed the study. AM and INO were responsible for data preparation. INO conducted analyses and produced the first draft of the manuscript. All authors critically commented on the drafts the manuscript and approved the final version for submission to the journal.

## Financial support

This research received no specific grant from any funding agency, commercial or not-for-profit sectors.

## Ethics approval

Approval for this study was obtained from the Office for Research Ethics Committees Northern Ireland (ORECNI), approval reference number 16/SC/0241. Informed consent from the subjects was not necessary because only anonymised data were released to the research team. The linked data were anonymised, held in the Administrative Data Research Centre Northern Ireland (ADRCNI) secure data environment within NISRA and made available to the research team for the purpose of this study.

## Declaration of competing interest

None.
